# Supplementary material for: Steering complex networks toward desired dynamics
Source: Sci Rep. 2020 Nov 27;10:20744. doi: 10.1038/s41598-020-77663-1 (PMC7695727; doi:10.1038/s41598-020-77663-1)
Supplement: Supplementary file 1 — Supplementary Information. [file 41598_2020_77663_MOESM1_ESM.pdf]

SUPPLEMENTARY INFORMATION:
Steering complex networks toward desired dynamics

Ricardo Gutiérrez,[1,*] Massimo Materassi,[2] Stefano Focardi,[2] and Stefano Boccaletti[2,3,4,5]

[1] *Complex Systems Interdisciplinary Group (GISC), Department of Mathematics, Universidad Carlos III de Madrid, 28911 Leganés, Madrid, Spain*
[2] *CNR, Institute of Complex Systems, Via Madonna del Piano 10, 50019 Florence, Italy*
[3] *Unmanned Systems Research Institute, Northwestern Polytechnical University, Xi'an 710072, China*
[4] *Moscow Institute of Physics and Technology (National Research University), 9 Institutskiy per., Dolgoprudny, Moscow Region, 141701, Russian Federation*
[5] *Universidad Rey Juan Carlos, Calle Tulipán, s/n, 28933 Móstoles, Madrid, Spain*

A. CORRELATIONS OF THE TARGETING SEQUENCE AND THE INFLUENCE INDEX FOR OTHER NETWORK TOPOLOGIES

As briefly mentioned in the main text, the targeting sequence is correlated with the influence index $k_{\mathrm{out}}/k_{\mathrm{in}}$ in the networks with uniform degree distributions considered in Fig. 1. And this is also the case with other networks topologies, such as Erdös-Rényi random graphs and Barabási-Albert scale-free networks. Results analogous to those of Fig. 1, with the same parameter choices, are shown Fig. A1, for the Erdös-Rényi case, and Fig. A2, for the Barabási-Albert scale-free networks.

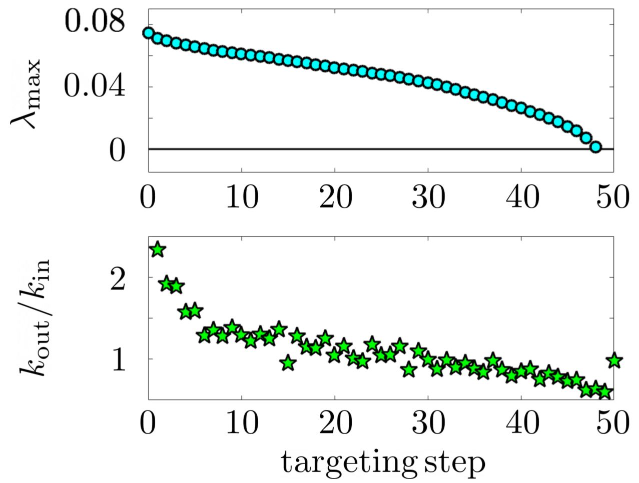

FIG. A1. **Controlling the dynamics of a mixed Erdös-Rényi random graph of $N = 50$ nonlinearly-coupled Rössler oscillators with intra-layer coupling $\sigma_1 = 0.01$ and inter-layer coupling $\sigma_2 = 1$.** (Top). Maximum Lyapunov exponent λ_{max} as a function of the targeting step. (Bottom) Influence index $k_{\mathrm{out}}/k_{\mathrm{in}}$ of the node that is pinned at each targeting step.

In these figures, the maximum Lyapunov exponent λ_{max} corresponding to the last step of the targeting sequence, which is negative and has a comparatively large absolute value, is not shown in the top panel for visibility reasons. With just 20 network realizations of size $N = 50$, one can clearly see that, except for minor fluctutations, the targeting sequence starts from nodes with high influence index and proceeds toward nodes with a smaller influence in the network.

While these results, as well as those in Fig. 1, correspond to an intra-layer coupling strength $\sigma_1 = 0.01$, increasing this value tends to reduce the correlation between targeting sequence and influence index, or even to destroy it altogether. For example, Fig. A3 shows results analogous to those in Fig. A1, for the Erdös-Rény case, but with a coupling strength $\sigma_1 = 0.05$, where only the first node in the targeting sequence seems to have a very high influence

* Correspondence should be addressed to: rigutier@math.uc3m.es

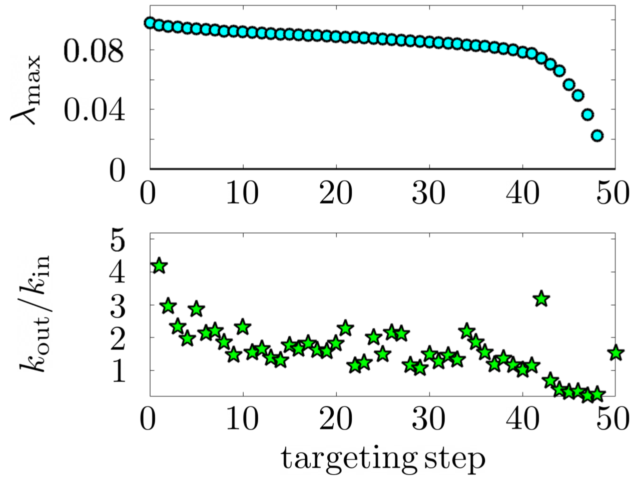

FIG. A2. **Controlling the dynamics of a mixed Barabási-Albert scale-free network of $N = 50$ nonlinearly-coupled Rössler oscillators with intra-layer coupling $\sigma_1 = 0.01$ and inter-layer coupling $\sigma_2 = 1$.** (Top). Maximum Lyapunov exponent $\lambda_{\rm max}$ as a function of the targeting step. (Bottom) Influence index $k_{\rm out}/k_{\rm in}$ of the node that is pinned at each targeting step.

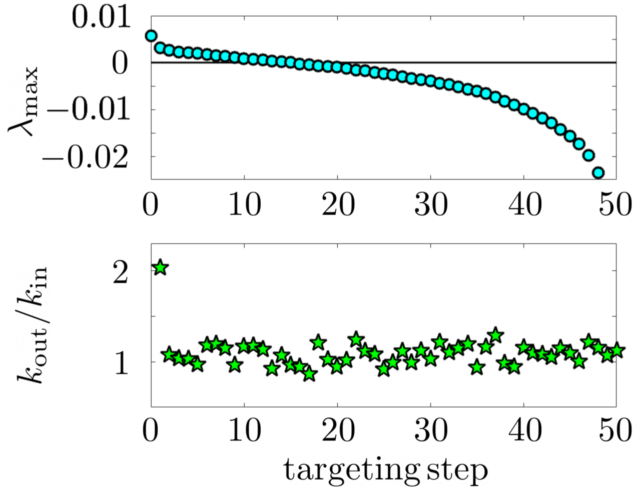

FIG. A3. **Controlling the dynamics of a mixed Erdös-Rényi random graph of $N = 50$ nonlinearly-coupled Rössler oscillators with intra-layer coupling $\sigma_1 = 0.05$ and inter-layer coupling $\sigma_2 = 1$.** (Top). Maximum Lyapunov exponent $\lambda_{\rm max}$ as a function of the targeting step. (Bottom) Influence index $k_{\rm out}/k_{\rm in}$ of the node that is pinned at each targeting step.

index $k_{\rm out}/k_{\rm in}$. Similarly, the network with uniform out-degree $k_{\rm out}$ and in-degree $k_{\rm in}$ distributions considered in the main text and Fig. 1, seems to show a complete lack of correspondence between the targeting sequence and the influence index ranking when the coupling strength is $\sigma_1 = 0.05$ (not shown). However, such results may well be related to the fact that for those networks, with such a high intra-layer coupling strength, very few targeting steps are required to achieve inter-layer synchronization. That might also be the reason why in Fig. A3, for the Erdös-Rény topology, the first node is in fact highly influential, because, by pinning just one node, the network is already very close to inter-layer synchronization. In fact, we do have some evidence that suggests that this could be the case: Fig. A4 shows results analogous to those in Fig. A2, for Barabási-Albert scale-free networks, but with a coupling strength $\sigma_1 = 0.05$, instead of $\sigma_1 = 0.01$, where a large number of nodes need to be pinned before inter-layer synchronization is attained. In that case, we do find a clear correlation between the targeting sequence and the influence index ranking.

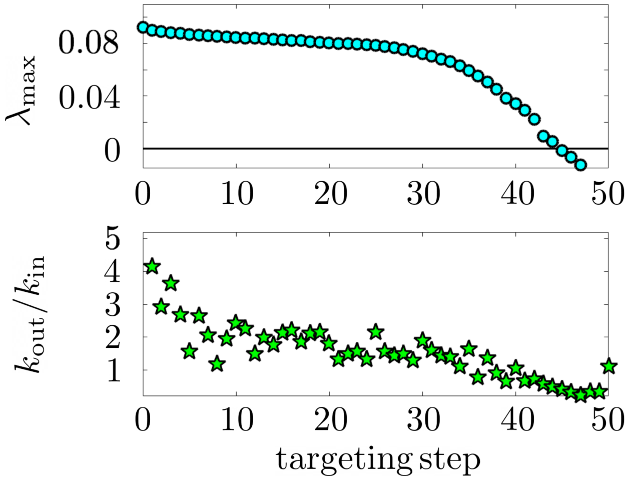

FIG. A4. **Controlling the dynamics of a mixed Barabási-Albert scale-free network of $N = 50$ nonlinearly-coupled Rössler oscillators with intra-layer coupling $\sigma_1 = 0.05$ and inter-layer coupling $\sigma_2 = 1$.** (Top). Maximum Lyapunov exponent $\lambda_{\max}$ as a function of the targeting step. (Bottom) Influence index $k_{\text{out}}/k_{\text{in}}$ of the node that is pinned at each targeting step.

B. TROPHIC WEB MODEL

The considered model mimics the behavior of an actual trophic web of large terrestrial vertebrates. It is inspired by holarctic ecosystems found in Asia, Europe and North America. It includes 12 dynamical variables, representing the densities of 12 species. The variables are:

- H_1, H_2 , H_3 and H_4, which refer to large herbivores, possibly different species of deer;
- H_6, which represents the population of an omnivorous mammal such as the wild boar;
- J_1 and J_3, which are the number densities of small herbivores, for instance the hare and the beaver;
- M_1 and M_2, which stand for the populations of two mesopredators, such as the wolverine and the fox;
- P_1, which is the number density of a large omnivorous species, in competition with the mesopredators, for instance the bear;
- P_2 and P_3, which refer to large predators, for instance the wolf and the lynx.

For simplicity, we assume that herbivores and generalist feeders (H_1, H_2, H_4, H_6, J_1, J_3, M_2 and P_1) follow a logistic growth:

$$\dot{X} = R\left(1 - \frac{X}{K}\right)X, \tag{B1}$$

while the mesopredator M_1 and the predators P_2 and P_3 undergo stand-alone equations of exponential decay:

$$Y = -\Delta Y. \tag{B2}$$

Notice that in Eqs. (B1) and (B2), R, K and Δ are all population-independent quantities. In the absence of other populations, Eq. (B1) gives rise to a growth dynamics which asymptotically reaches the value $X = K$; while Eq. (B2) will lead to extinction of the species on a time scale of the order of $\frac{1}{\Delta}$. This means that populations H_1, H_2, H_4, H_6, J_1, J_3, M_2 and P_1 may be sustained "by the environment" in the absence of the other species, while M_1, P_2 and P_3 will starve if left alone. In other words, populations M_1, P_2 and P_3 can live only when the other species are present, while populations H_1, H_2, H_4, H_6, J_1, J_3, M_2 and P_1 are fed by resources which are not included in the trophic web model.

As for the growth rate coefficient R, this is a constant quantity for all species except for the omnivorous H_6, for which one has instead

$$R_{H_6}(t) = R_{0H_6} + W_{H_6}\sin^2\left(\frac{\omega t}{2}\right). \tag{B3}$$

The above Equation accounts for a time-dependent, periodic resource supply from the environment to the population H_6 (for instance it may refer to the periodic hyper-production of acorn, if H_6 describes wild boars).

The interactions between populations may be of two kinds: either prey-predator-like, or competitive.

If a population Y preys on the population X, this brings a term $\dot{X}_Y^{\text{pred}}$ in the equation for X, and a term $\dot{Y}_X^{\text{pred}}$ in that for Y. The evolution equations describe the case of *satiable predators* [1], and take the form

$$\begin{cases} \dot{X}_Y^{\text{pred}} = -f_{XY}\left(X\right)Y,\ \dot{Y}_X^{\text{pred}} = C_{XY}f_{XY}\left(X\right)Y, \\ \\ f_{XY}\left(X\right) = \dfrac{A_{XY}X}{B_{XY}+Y}. \end{cases} \tag{B4}$$

The coefficients A_{XY}, B_{XY} and C_{XY} are all positive constants: if $\dot{X}_Y^{\text{pred}}$ is negative, then $\dot{Y}_X^{\text{pred}}$ is larger than zero, as predation is advantageous for the predator and disadvantageous for the prey.

On the other hand, competitive interactions lead to two negative terms in the equations of the species X and Y:

$$\dot{X}_Y^{\text{comp}} = -\alpha_{XY}XY,\ \dot{Y}_X^{\text{comp}} = -\alpha_{YX}XY. \tag{B5}$$

The two quantities α_{XY} and α_{YX} are both positive, and not necessarily equal: indeed, competition may be more disadvantageous for one species than for the other one.

Therefore, the whole trophic web can be described by the following system of differential equations:

Equation for H_1:

$$\begin{gathered} \dot{H}_1 = R_{H_1}\left(1-\frac{H_1}{K_{H_1}}\right)H_1+ \\ -\alpha_{13}H_1H_3-\alpha_{12}H_2H_1-\alpha_{14}H_1H_4-\alpha_{16}H_1H_6+ \\ -f_{H_1M_1}\left(H_1\right)M_1-f_{H_1M_2}\left(H_1\right)M_2+ \\ -f_{H_1P_2}\left(H_1\right)P_2-f_{H_1P_3}\left(H_1\right)P_3. \end{gathered} \tag{B6}$$

Equation for H_2:

$$\begin{gathered} \dot{H}_2 = R_{H_2}\left(1-\frac{H_2}{K_{H_2}}\right)H_2+ \\ -\alpha_{21}H_1H_2-\alpha_{23}H_2H_3-\alpha_{24}H_2H_4-\alpha_{26}H_2H_6+ \\ -f_{H_2M_1}\left(H_2\right)M_1-f_{H_2M_2}\left(H_2\right)M_2+ \\ -f_{H_2P_2}\left(H_2\right)P_2-f_{H_2P_3}\left(H_2\right)P_3. \end{gathered} \tag{B7}$$

Equation for H_3:

$$\begin{gathered} \dot{H}_3 = R_{H_3}\left(1-\frac{H_3}{K_{H_3}}\right)H_3+ \\ -\alpha_{31}H_1H_3-\alpha_{32}H_2H_3-\alpha_{34}H_3H_4-\alpha_{36}H_3H_6+ \\ -f_{H_3M_1}\left(H_3\right)M_1-f_{H_3M_2}\left(H_3\right)M_2+ \\ -f_{H_3P_2}\left(H_3\right)P_2-f_{H_3P_3}\left(H_3\right)P_3. \end{gathered} \tag{B8}$$

Equation for H_4:

$$\begin{gathered} \dot{H}_4 = R_{H_4}\left(1-\frac{H_4}{K_{H_4}}\right)H_4+ \\ -\alpha_{41}H_1H_4-\alpha_{42}H_2H_4-\alpha_{43}H_3H_4-\alpha_{46}H_4H_6+ \\ -f_{H_4M_2}\left(H_4\right)M_2+ \\ -f_{H_4P_2}\left(H_4\right)P_2-f_{H_4P_3}\left(H_4\right)P_3. \end{gathered} \tag{B9}$$

Equations for H_6:

$$\dot{H}_6 = \left[R_{0H_6} + W_{H_6}\sin^2\left(\frac{\omega t}{2}\right)\right]\left(1 - \frac{H_6}{K_{H_6}}\right)H_6 + \\ -\alpha_{61}H_1H_6 - \alpha_{62}H_2H_6 - \alpha_{63}H_3H_6 + \\ -f_{H_6M_2}\left(H_6\right)M_2 + \\ -f_{H_6P_2}\left(H_6\right)P_2 - f_{H_6P_3}\left(H_6\right)P_3 \tag{B10}$$

Equations for J_1:

$$\dot{J}_1 = R_{J_1}\left(1 - \frac{J_1}{K_{J_1}}\right)J_1 + \\ -f_{J_1M_1}\left(J_1\right)M_1 - f_{J_1M_2}\left(J_1\right)M_2 + \\ -f_{J_1P_3}\left(J_1\right)P_3. \tag{B11}$$

Equations for J_3:

$$\dot{J}_3 = R_{J_3}\left(1 - \frac{J_3}{K_{J_3}}\right)J_3 + \\ -f_{J_3M_1}\left(J_3\right)M_1 - f_{J_3M_2}\left(J_3\right)M_2. \tag{B12}$$

Equations for M_1:

$$\dot{M}_1 = -\Delta_{M_1}M_1 + \\ -\beta_{11}M_1P_1 - \beta_{12}M_1P_2 - \beta_{13}M_1P_3 + \\ +\left[C_{H_1M_1}f_{H_1M_1}\left(H_1\right) + C_{H_2M_1}f_{H_2M_1}\left(H_2\right) + \\ +C_{H_3M_1}f_{H_3M_1}\left(H_3\right) + \\ +C_{J_1M_1}f_{J_1M_1}\left(J_1\right) + C_{J_3M_1}f_{J_3M_1}\left(J_3\right)\right]M_1. \tag{B13}$$

Equations for M_2:

$$\dot{M}_2 = R_{M_2}\left(1 - \frac{M_2}{K_{M_2}}\right)M_2 + \\ -\beta_{21}M_2P_1 - \beta_{22}M_2P_2 - \beta_{23}M_2P_3 + \\ +\left[C_{H_1M_2}f_{H_1M_2}\left(H_1\right) + C_{H_2M_2}f_{H_2M_2}\left(H_2\right) + \\ +C_{H_3M_2}f_{H_3M_2}\left(H_3\right) + C_{H_4M_2}f_{H_4M_2}\left(H_4\right) + \\ +C_{H_6M_2}f_{H_6M_2}\left(H_6\right) + \\ +C_{J_1M_2}f_{J_1M_2}\left(J_1\right) + C_{J_3M_2}f_{J_3M_2}\left(J_3\right)\right]M_2. \tag{B14}$$

Equations for P_1:

$$\dot{P}_1 = R_{P_1}P_1\left(1 - \frac{P_1}{K_{P_1}}\right) + \\ -\gamma_{11}P_1M_1 - \gamma_{12}P_1M_2. \tag{B15}$$

Equations for P_2:

$$\begin{gathered}\dot{P}_2 = -\Delta_{P_2} P_2 + \\ -\gamma_{21} P_2 M_1 - \gamma_{22} P_2 M_2 + \\ + \left[C_{H_1 P_2} f_{H_1 P_2} (H_1) + C_{H_2 P_2} f_{H_2 P_2} (H_2) + \right. \\ + C_{H_3 P_2} f_{H_3 P_2} (H_3) + \\ \left. + C_{H_4 P_2} f_{H_4 P_2} (H_4) + C_{H_6 P_2} f_{H_6 P_2} (H_6) \right] P_2.\end{gathered} \tag{B16}$$

Equations for P_3:

$$\begin{gathered}\dot{P}_3 = -\Delta_{P_3} P_3 + \\ -\gamma_{31} P_3 M_1 - \gamma_{32} P_3 M_2 + \\ + \left[C_{J_1 P_3} f_{J_1 P_3} (J_1) + C_{H_1 P_3} f_{H_1 P_3} (H_1) + \right. \\ + C_{H_2 P_3} f_{H_2 P_3} (H_2) + C_{H_3 P_3} f_{H_3 P_3} (H_3) + \\ \left. + C_{H_4 P_3} f_{H_4 P_3} (H_4) + C_{H_6 P_3} f_{H_6 P_3} (H_6) \right] P_3.\end{gathered} \tag{B17}$$

For simplicity, the coefficients introduced as α_{XY} and α_{YX} in Eq.(B5) have been renamed (all over the equations) as β and γ, respectively.

The parameters appearing in the model are assigned as follows.

Parameters for H_1:

$$R_{H_1} = 0.5,\ K_{H_1} = 50,$$

$$A_{H_1 M_1} = \frac{4}{15},\ B_{H_1 M_1} = 20,\ A_{H_1 M_2} = \frac{7}{16},\ B_{H_1 M_2} = 20,$$

$$A_{H_1 P_2} = \frac{5}{18},\ B_{H_1 P_2} = 20,\ A_{H_1 P_3} = \frac{18}{89},\ B_{H_1 P_3} = 15,$$

$$\alpha_{12} = 2 \times 10^{-4},\ \alpha_{13} = 3 \times 10^{-4},\ \alpha_{14} = 5 \times 10^{-4},\ \alpha_{16} = 10^{-5}.$$

Parameters for H_2:

$$R_{H_2} = 0.25,\ K_{H_2} = 15,$$

$$A_{H_2 M_1} = 0.05,\ B_{H_2 M_1} = 8,\ A_{H_2 M_2} = 0.075,\ B_{H_2 M_2} = 8,$$

$$A_{H_2 P_2} = 0.1,\ B_{H_2 P_2} = 10,\ A_{H_2 P_3} = \frac{1}{34},\ B_{H_2 P_3} = 10,$$

$$\alpha_{21} = 10^{-6},\ \alpha_{23} = 3 \times 10^{-5},\ \alpha_{24} = 5 \times 10^{-4},\ \alpha_{26} = 10^{-6}.$$

Parameters for H_3:

$$R_{H_3} = 0.45,\ K_{H_3} = 2,$$

$$A_{H_3M_1} = 10^{-4},\ B_{H_3M_1} = 1,\ A_{H_3M_2} = 10^{-5},\ B_{H_3M_2} = 1,$$

$$A_{H_3P_2} = 0.06,\ B_{H_3P_2} = 1.5,\ A_{H_3P_3} = \frac{2}{325},\ B_{H_3P_3} = 1.5,$$

$$\alpha_{31} = 10^{-6},\ \alpha_{32} = 10^{-4},\ \alpha_{34} = 10^{-6},\ \alpha_{36} = 10^{-6}.$$

Parameters for H_4 :

$$R_{H_4} = 0.3,\ K_{H_4} = 40,$$

$$A_{H_4M_2} = 0.5,\ B_{H_4M_2} = 25,\ A_{H_4P_2} = \frac{7}{30},\ B_{H_4P_2} = 15,$$

$$A_{H_4P_3} = 0.075,\ B_{H_4P_3} = 15,\ \alpha_{41} = 10^{-4},\ \alpha_{42} = 2 \times 10^{-4},$$

$$\alpha_{43} = 10^{-5},\ \alpha_{46} = 10^{-4}.$$

Parameters for H_6:

$$R_{H_6} = 0.8, W_{H_6} = 5, K_{H_6} = 40,$$

$$A_{H_6P_2} = 0.5,\ B_{H_6P_2} = 20,\ A_{H_6P_3} = 0.3,\ B_{H_6P_3} = 15,$$

$$A_{H_6M_2} = 0,\ B_{H_6M_2} = 20,$$

$$\alpha_{61} = 10^{-5},\ \alpha_{62} = 10^{-5},\ \alpha_{63} = 10^{-5}.$$

Parameters for J_1:

$$R_{J_1} = 0.8,\ K_{J_1} = 100,$$

$$A_{J_1M_1} = 2.2,\ B_{J_1M_1} = 50,\ A_{J_1M_2} = 0.1,\ B_{J_1M_2} = 50,$$

$$A_{J_1P_3} = 0.5,\ B_{J_1P_3} = 90.$$

Parameters for J_3:

$$R_{J_3} = 1.2,\ K_{J_3} = 15,$$

$$A_{J_3M_1} = 1,\ B_{J_3M_1} = 5,\ A_{J_3M_2} = 0.1,\ B_{J_3M_2} = 5.$$

Parameters for M_1:

$$\Delta_{M_1} = \frac{1}{15},\ \beta_{11} = 10^{-3},\ \beta_{12} = 5 \times 10^{-3},\ \beta_{13} = 10^{-4},$$

$$C_{H_1M_1} = 0.25,\ C_{H_2M_1} = 0.35,\ C_{H_3M_1} = 0.4,$$

$$C_{J_1M_1} = 0.05,\ C_{J_3M_1} = 0.05.$$

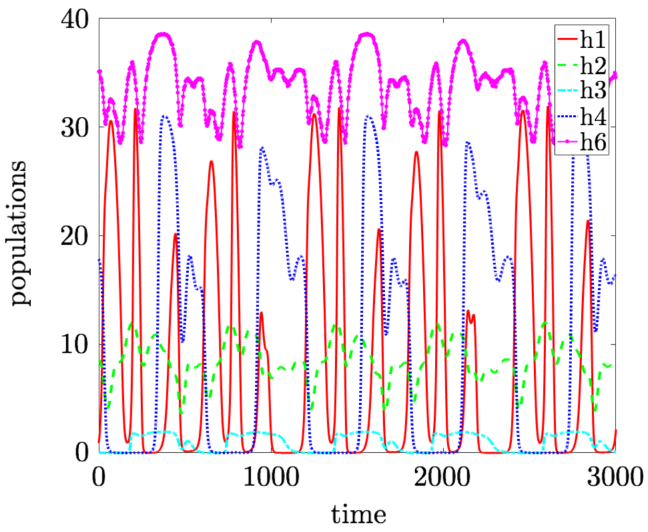

FIG. B1. Time evolution of the populations of large herbivores (H1-H4) and of the omnivorous mammal (H6). The color code is specified in the legend.

Parameters for M_2:

$$R_{M_2} = 1.2,\ K_{M_2} = 5,$$

$$\beta_{21} = 10^{-3},\ \beta_{22} = 5 \times 10^{-3},\ \beta_{23} = 10^{-4},$$

$$C_{H_1M_2} = 0.3,\ C_{H_2M_2} = 0.35,\ C_{H_3M_2} = 0.35,$$

$$C_{H_4M_2} = 0.35,\ C_{H_6M_2} = 0.3,$$

$$C_{J_1M_2} = 0.05,\ C_{J_3M_2} = 0.05.$$

Parameters for P_1:

$$R_{P_1} = 0.25,\ K_{P_1} = 0.1,$$

$$\gamma_{11} = 10^{-6},\ \gamma_{12} = 10^{-6}.$$

Parameters for P_2:

$$\Delta_{P_2} = 0.3,\ \gamma_{21} = 10^{-6},\ \gamma_{22} = 10^{-6},\ C_{H_1P_2} = 0.4,$$

$$C_{H_2P_2} = 0.7,\ C_{H_3P_2} = 0.8,\ C_{H_4P_2} = 0.6,\ C_{H_6P_2} = 0.6.$$

Parameters for P_3:

$$\Delta_{P_3} = 0.1,\ \gamma_{31} = 10^{-6},\ \gamma_{32} = 10^{-6},$$

$$C_{J_1P_3} = 0.05,\ C_{H_1P_3} = 0.3,\ C_{H_2P_3} = 0.3,$$

$$C_{H_3P_3} = 0.4,\ C_{H_4P_3} = 0.3,\ C_{H_6P_3} = 0.3.$$

With this parameter choice, the twelve species display chaotic oscillations, with a maximum Lyapunov exponent $\Lambda_{\max} \simeq 0.0014$, which was calculated by use of the method introduced in Ref. [2]. In Fig. B1, irregular oscillations are

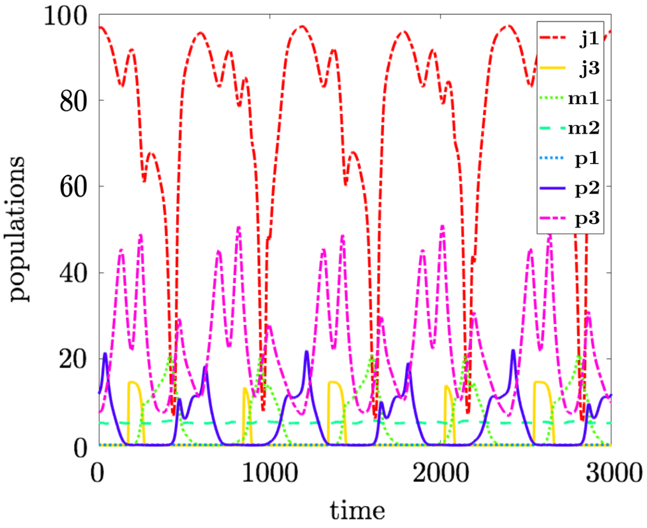

FIG. B2. Time evolution of the populations of small herbivores (J1 and J3), mesopredators (M1 and M2), a large omnivorous species (P1) and large predators (P2 and P3). The color code is specified in the legend.

reported which characterize the evolution of the large herbivors (H1-H4) and of the omnivorous mammal (H6). The remaining species, including small herbivores (J1 and J3), mesopredators (M1 and M2), a large omnivorous species (P1) and large predators (P2 and P3), are shown in Fig. B2.

[1] Materassi, M., Innocenti, G., Berzi, D. & Focardi, S. Kleptoparasitism and complexity in a multi-trophic web. *Ecological Complexity* **29**, 49–60 (2017).

[2] Benettin, G., Galgani, L., Giorgilli, A. & Strelcyn, J.-M. Lyapunov Characteristic Exponents for smooth dynamical systems and for Hamiltonian systems; a method for computing all of them. part 2: Numerical application. *Meccanica* **15**, 21–30 (1980).
